# Locomotor learning in infants at high risk for cerebral palsy: A study protocol

**DOI:** 10.3389/fped.2023.891633

**Published:** 2023-02-23

**Authors:** Laura A. Prosser, Julie Skorup, Samuel R. Pierce, Abbas F. Jawad, Andrew H. Fagg, Thubi H. A. Kolobe, Beth A. Smith

**Affiliations:** ^1^ Division of Rehabilitation Medicine, The Children’s Hospital of Philadelphia, Philadelphia, PA, United States; ^2^ Department of Pediatrics, Perelman School of Medicine, University of Pennsylvania, Philadelphia, PA, United States; ^3^ Department of Physical Therapy, The Children’s Hospital of Philadelphia, Philadelphia, PA, United States; ^4^ Division of General Pediatrics, The Children’s Hospital of Philadelphia, Philadelphia, PA, United States; ^5^ Department of Computer Science, University of Oklahoma, Norman, OK, United States; ^6^ Institute for Biomedical Engineering, Science and Technology, University of Oklahoma, Norman, OK, United States; ^7^ Department of Rehabilitation Science, University of Oklahoma Health Sciences Center, Oklahoma, OK, United States; ^8^ Developmental Neuroscience and Neurogenetics Program, The Saban Research Institute, Children’s Hospital Los Angeles, Los Angeles, CA, United States; ^9^ Division of Developmental-Behavioral Pediatrics, Children's Hospital Los Angeles, Los Angeles, CA, United States; ^10^ Department of Pediatrics, Keck School of Medicine, University of Southern California, Los Angeles, CA, United States

**Keywords:** cerebral palsy, rehabilitation, physical therapy, infant, motor learning, robotics, crawling

## Abstract

**Background:**

Physical disability in individuals with cerebral palsy (CP) creates lifelong mobility challenges and healthcare costs. Despite this, very little is known about how infants at high risk for CP learn to move and acquire early locomotor skills, which set the foundation for lifelong mobility. The objective of this project is to characterize the evolution of locomotor learning over the first 18 months of life in infants at high risk for CP. To characterize how locomotor skill is learned, we will use robotic and sensor technology to provide intervention and longitudinally study infant movement across three stages of the development of human motor control: early spontaneous movement, prone locomotion (crawling), and upright locomotion (walking).

**Study design:**

This longitudinal observational/intervention cohort study (ClinicalTrials.gov Identifier: NCT04561232) will enroll sixty participants who are at risk for CP due to a brain injury by one month post-term age. Study participation will be completed by 18 months of age. Early spontaneous leg movements will be measured monthly from 1 to 4 months of age using inertial sensors worn on the ankles for two full days each month. Infants who remain at high risk for CP at 4 months of age, as determined from clinical assessments of motor function and movement quality, will continue through two locomotor training phases. Prone locomotor training will be delivered from 5 to 9 months of age using a robotic crawl training device that responds to infant behavior in real-time. Upright locomotor training will be delivered from 9 to 18 months of age using a dynamic weight support system to allow participants to practice skills beyond their current level of function. Repeated assessments of locomotor skill, training characteristics (such as movement error, variability, movement time and postural control), and variables that may mediate locomotor learning will be collected every two months during prone training and every three months during upright training.

**Discussion:**

This study will develop predictive models of locomotor skill acquisition over time. We hypothesize that experiencing and correcting movement errors is critical to skill acquisition in infants at risk for CP and that locomotor learning is mediated by neurobehavioral factors outside of training.

**Project Number** 1R01HD098364-01A1.

**ClinicalTrials.gov Identifier:** NCT04561232

## 1. Introduction

Cerebral palsy (CP) is the most common physical disability in children (1, 2). Perinatal brain injury, including periventricular leukomalacia, intraventricular hemorrhage and hypoxic ischemic encephalopathy, increase the risk of CP (3). CP limits full inclusion and integration in society throughout the lifespan. The degree of functional limitations and life participation restriction are predicted by the degree of motor disability, which varies widely from poor motor coordination and mobility to full dependence on others for care (4). Mobility limitations are a result of impairment in the development of motor control during the first two years of life; a critical period for neuroplasticity in the motor control centers of the brain (5, 6). Despite this, very little research effort has been directed towards mitigating disability in the early years of life, when the foundation for lifespan functional mobility is built.

Children with CP demonstrate failures and discontinuities in skill acquisition, poor retention or sustainability of short-term gains (7–9) and poor transferability of learning from one skill to another (10). This is in contrast to children with typical development (TD), whose early mobility skills serve a scaffolding role to later and higher function. For example, approximately 90% of infants who walk by 18 months of age first learn to crawl (11). Some similarities have also been observed between gait patterns and crawling speed (12). These relationships are tenuous in children with CP (13) with many crawlers who never learn to walk and many walkers who never crawled. Intervention studies have also highlighted motor learning discontinuities. For example, treadmill training studies have reported improved stepping quality but no improvement in walking onset in infants with CP (8, 14) raising questions about the retention and transfer of the training. Similarly, findings from constraint induced therapy show inconsistent learning retention and transfer to other functional skills (9, 10). Typical motor skill development is characterized by high practice repetitions (11, 15), error-based learning (15), movement variability (16, 17), early postural stability (7, 18), and movement efficiency (19). In contrast to their peers, children with CP demonstrate a lack of self-initiated locomotor experiences during infancy, which interferes with early exploration of the environment and critical experiences for cognitive development, such as autonomy, visual-motor integration, problem solving, and social interaction (20–24). This body of work underscores the need to identify the mechanisms for motor learning, retention, and transfer in CP, which are not yet understood, but are key to developing interventions whose effects are long-lasting and foster transfer to other motor skills later in development.

### 1.1. Rationale for robotic and sensor technology

Robotic and sensor technology have the potential to provide novel types of information about how infants with CP learn or fail to locomote and may inform early prognosis. This is particularly key as infants cannot follow training commands. Embedding robotic and sensor technology in functional training paradigms offers a unique opportunity to provide immediate response and feedback that are contingent on the infant's actions and measure motor learning in real time. This information can be used to make predictions about learning retention and transfer of skills. Traditional studies have relied on observations or standardized scales that evaluate milestone achievement. A major limitation of these is the inability to capture the movement variability that characterizes movement learning in children, particularly those with CP, and to do so in real time.

We have developed a protocol to investigate the longitudinal progression of locomotor learning across three key stages in the development of motor control in infants with CP—early spontaneous movement (1–4 months of age), prone locomotion (5–9 months), and upright locomotion (9–18 months). Phase one is the observation of early spontaneous leg movements using wearable sensors. Early spontaneous infant leg movement is related to the later locomotor skill of walking and is different between infants at risk for developmental disabilities and infants with typical development (25, 26). Phase two is the intervention phase including prone and upright locomotor training. Prone locomotion is when the infant moves using upper and lower limbs with the anterior aspect of the trunk aligned to face the floor- this includes crawling and creeping on hands and knees. Upright locomotion is when the infant moves using lower limbs only with the trunk aligned vertically in space—this includes knee walking and walking.

Common across all three stages is the collection of comprehensive robotic, sensor, and behavioral data about how infants at risk for CP move. Intervention is introduced early, before an infant's milestones emerge or fail to emerge because the first two years of life is recognized as a critical period of neuroplasticity for motor control centers in the brain (27). This neuromotor plasticity is activity-dependent and more robust in early, as compared to later years (5, 6), and motor and cognitive gains are greater from earlier intervention (28). The intervention is designed to provide reinforcement (29) and error-based learning (30), two motor learning mechanisms that have been shown to improve skill learning in adults (31, 32), but that have only been minimally studied in skill learning in infants and toddlers (33, 34). Our staged longitudinal approach will enable the study of the evolution of locomotor learning across the development of motor control, providing fundamental missing information about mechanisms for motor learning in CP.

The standard clinical care for infants at risk for CP is varied; it can range from no rehabilitation services to several sessions of physical and occupational therapy per week (35). The initiation of therapy services often does not occur until after the child has missed major motor milestones (i.e., sitting, crawling, walking). This is often when the child is closer to one year of age, thus missing foundational movement experience during critical periods of high neuroplasticity. Further, it is rare that standard of care therapy would utilize robotic technology as a tool to deliver the intervention. There are several reasons for this—few technologies exist for this young population, and those that do are usually clinic-based and cannot be transported to the patient's home. We propose robotic technology in this work because it can help deliver the intervention in a standardized way, allow error-based learning and it can quantify various aspects of learning that cannot be measured from conventional approaches to motor training.

The goal of this study is to explore determinants of locomotor skill acquisition (demonstration of a new skill), retention (repeated demonstration or use of a skill over time), and transfer (use of a skill in new contexts or environments) in infants at high risk for CP. Locomotor skills are activities that allow an infant to move independently from one place to another. We are exploring the key factors that allow infants to learn and keep new locomotor skills. Our central hypothesis is that experiencing error is a key mechanism for locomotor learning, but that this learning is mediated by other neurobehavioral factors, including early spontaneous movement, cognition, and motivation to move.

The first aim of this study is to characterize patterns of error-based learning during the acquisition of locomotor skill in infants at high risk for CP. We will examine the relationships between error and locomotor skill acquisition over time. We expect that experiencing and correcting movement errors is critical to skill acquisition in infants at risk for CP. Specifically, we hypothesize that a) total amount of error and error rate (per distance travelled) will be positively associated with the acquisition of locomotor skill, b) after the maximum amount of error is reached, the strength of the relationship between error and locomotor proficiency will decrease (i.e., error will stabilize or diminish while locomotor skill continues to improve), and c) the relationship between error and skill acquisition will be similar for the two locomotor skills (prone and upright locomotion).

The second aim is to develop a predictive model of locomotor skill retention in infants at high risk for CP based on training characteristics and moderators. We will examine the contribution of training characteristics (movement time, level of postural control, and motor variability) to locomotor learning, and how learning is mediated by neurobehavioral factors outside of training (cognition, early spontaneous movement behavior, and motivation to move). We will develop comprehensive models of training predictors and mediators for prone and upright locomotor learning.

The third aim is to determine locomotor learning strategies in infants at high risk for CP that transfer from prone to upright locomotion. Our preliminary findings have shown that infants at risk for CP, like typically developing infants, are capable of using robotic, reinforcement-based feedback to engage in task-specific learning, but unlike their typically developing peers, show inconsistent learning transfer of locomotor skills from one context to another. Our pilot studies have focused on one skill and were time limited. We expect to identify critical thresholds of error and other training characteristics from prone locomotor training above which greater upright locomotor learning is likely.

## 2. Methods and analysis

### 2.1. Study design

**Figure 1:**
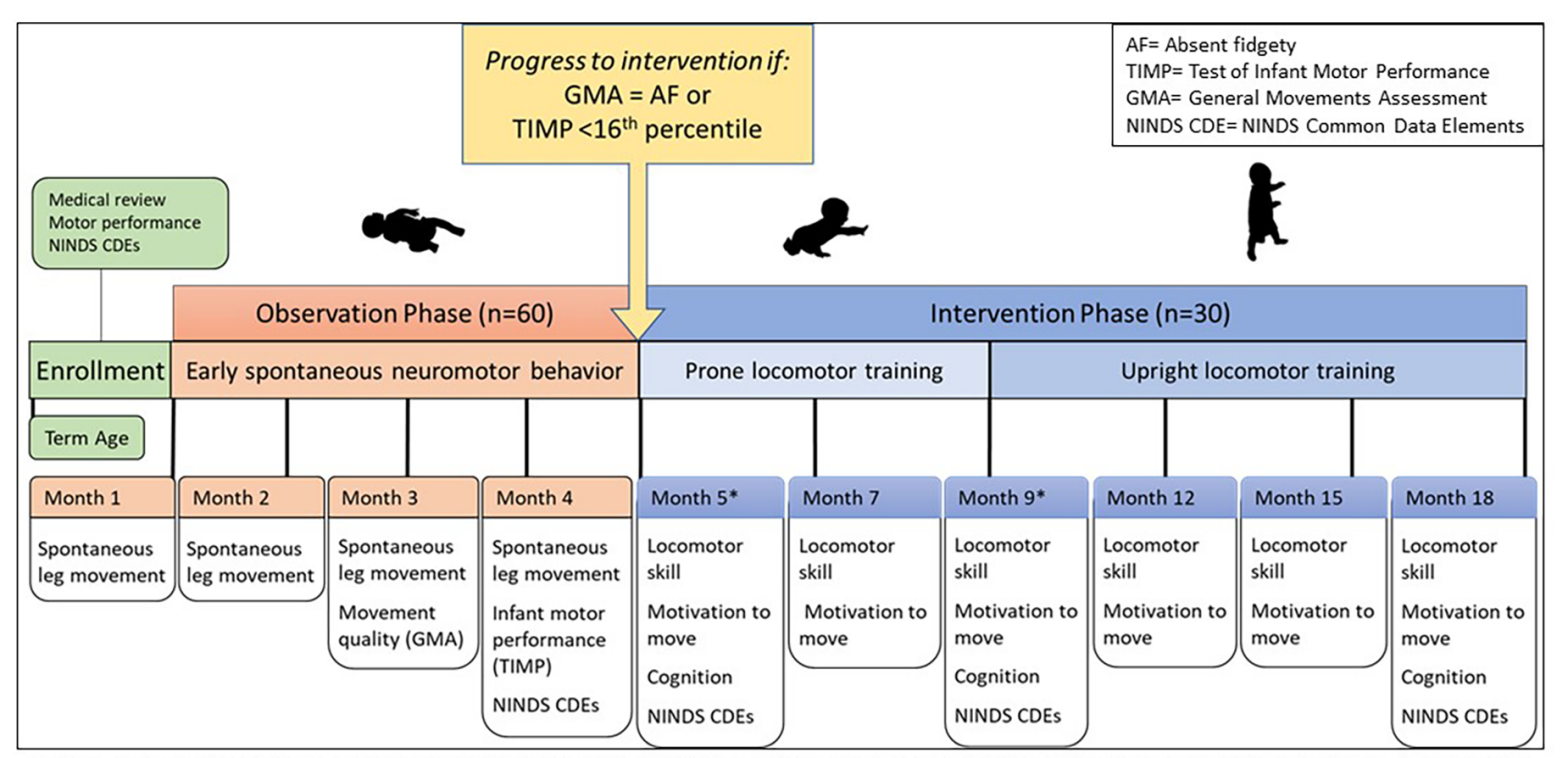
Longitudinal study design. Timeline is based on infant post-term age. Infants at high risk for CP be enrolled by one month post-term age. Data collected at each month time point is indicated. Early spontaneous movement will be measured during observation phase (1–4 months of age). Infants who remain at high risk for CP by Month 4 will continue to intervention phase (5–18 months of age). Prone locomotor training will be delivered from 5 to 9 months of age, followed by upright locomotor training will be delivered from 5 to 9 months of age, followed by upright locomotor training phase from 9 to 18 months of age. *Months 5 and 9 will be serve as baseline for prone and upright training phases, respecively.

This clinical trial is a longitudinal observational and interventional cohort study to characterize locomotor learning in infants at high risk for CP. Repeated measures of motor behavior and performance will characterize locomotor learning across the first 18 months of life from early spontaneous movements through the ages when prone and upright locomotion skills are achieved and mastered by the vast majority of typically developing infants but rarely by infants with CP. Sixty infants with early brain injury will be enrolled by one-month post-term age and complete Phase 1, an observation phase including leg movement monitoring for two consecutive days each month, from Months 1–4. Those who remain at high risk for CP at Month 4 will continue to Phase 2, an intervention phase including established crawling and walking training protocols to encourage locomotor learning, from Months 5–18. We anticipate that 30 participants will proceed to Phase 2. Participants will follow the study timeline based on post-term age. See Figure 1 for study design.

### 2.2. Study setting

This is a multi-site study with The Children's Hospital of Philadelphia (CHOP) being the primary site for participant recruitment, enrollment and data collection. Recruitment and Phase 1 data collection may also occur at the nearby Hospital of the University of Pennsylvania (HUP). Research team members from The University of Oklahoma Health Sciences Center and University of Oklahoma will develop the robotic and sensor technology, training protocol and data analysis plan for prone locomotion. A research team from the Children's Hospital Los Angeles will develop the data acquisition protocol and data analysis plan for the observation of infant leg movements. All sites will contribute to the training of CHOP study staff, fidelity monitoring and data analysis. Recruitment, intervention and data collection will occur at CHOP, HUP or in participants' homes or childcare environments. The location of study visits will be determined by the infants' disposition (inpatient or home/outpatient), caregiver preference, and visit type (upright locomotor training sessions will all occur at CHOP). CHOP's Institutional Review Board (IRB) is the IRB of record.

### 2.3. Participant criteria

We will enroll 60 infants who are at risk for CP. Inclusion criteria are: birth to 6 weeks post-term age; history of an early brain injury associated with high risk for CP, including periventricular leukomalacia, hypoxic-ischemic encephalopathy, intraventricular hemorrhage, hydrocephalus, stroke, neonatal seizures, or intracranial cystic lesion; and caregivers able to commit to study visits. Exclusion criteria are: a known genetic condition unrelated to CP, congenital abnormality and poor prognosis for survival, as determined by the medical team. Enrolled infants will be withdrawn from the trial if an excluding genetic condition, congenital abnormality or uncontrolled seizures are identified after enrollment. Participants will continue to receive their typical medical and rehabilitation care throughout the duration of the study.

### 2.4. Sample size estimation

Sixty infants will complete an observation phase for early spontaneous neuromotor behavior from Months 1–4 (Phase 1). We expect 30 to continue through locomotor training intervention phase from Months 5–18 (Phase 2). We expect the retention rate to be 80% or higher, resulting in 24 infants with evaluable datasets. This in-depth longitudinal study is not powered to achieve specific significance testing, but rather to estimate effect sizes related to the multidimensional complex relationships between training characteristics, locomotor skill, and potential mediating characteristics. A sample size of 24 infants with full data sets produces a two-sided 95% confidence interval for estimating changes in outcomes measured at two time points (for example Months 5 and 9 for prone locomotor training, and Months 9 and 18 for upright locomotor training) with a width equal to 0.76, 0.63, and 0.39 when the sample correlation is assumed to be equal to 0.25, 0.50, and 0.75, respectively. Similarly, a two-sided 95% CI with a width equal to 0.70 or 0.63 will be produced when the estimated Pearson's correlation between paired of outcomes (for example error rate and motor function) is 0.4 or 0.5, respectively. Finally, a sample size of 24 achieves 80% power to detect an R2 of 0.43 attributed to predicting motor function outcome in a regression model with 5 independent variables, using an F-Test with a significance level (alpha) of 0.05.

### 2.5. Phase 1

**Figure 2:**
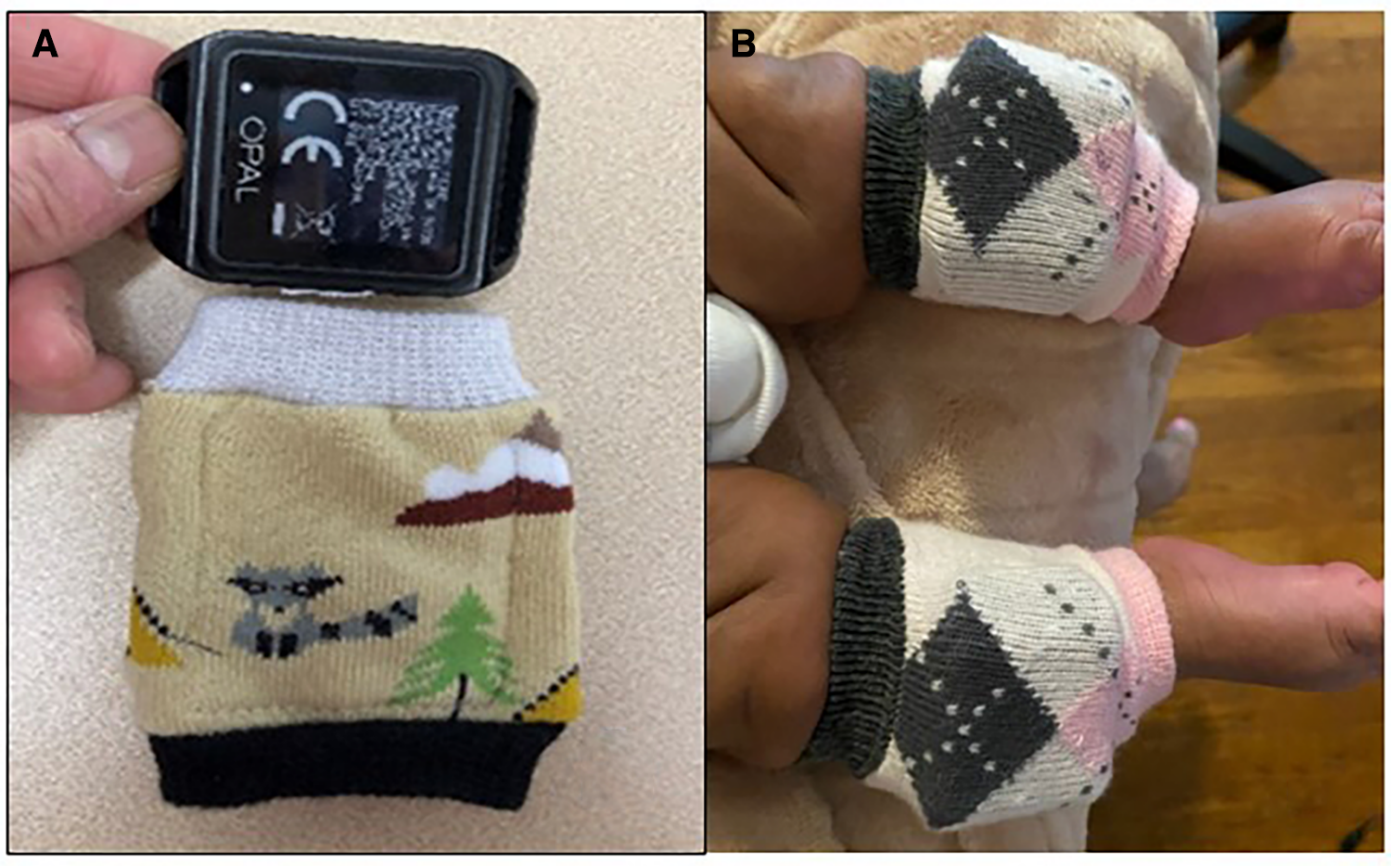
(**A**) Wearable sensor (top) and leg warmer with internal pocket to hold sensor (bottom). (**B**) Infant wearing sensors in leg warmers.

During the observation phase, monthly recording of spontaneous leg movements will begin at Month 1 and continue through Month 4. Infants will wear wireless inertial sensors on their ankles (Opal, APDM Inc, Portland, Oregon, United States.) for at least eight hours for two consecutive days, secured by custom leg warmers with a pocket to hold the sensor in place (see Figure 2). Two consecutive days is a sufficient and optimal amount of time to demonstrate an infant's typical daily performance while balancing burden on the infant/caregiver (36). If the infant is home, research staff will visit the home on day 1 of data collection to teach the caregivers how to place the sensors in the morning, remove them at bedtime, and charge them overnight. If the infant is still in the hospital, research staff will place and remove the sensors and coordinate with nursing staff to avoid interfering with clinical care.

### 2.6. Progression to phase 2

The determination if the infant will continue to study treatment phase is made using the General Movements Assessments (GMA) and the Test of Infant Motor Performance (TIMP). The GMA classifies the infant's spontaneous movement quality in the supine position through visual observation by a trained reviewer. The reliability and validity of the GMA have been reported in infants (37, 38)**.** All GMA reviewers will have completed the Basic Course offered by the General Movements Trust (39). Each GMA video will be reviewed by two reviewers. If the reviewers are not in agreement, a third reviewer will be asked to adjudicate the video. The GMA will first occur at Month 3 to evaluate for presence of fidgety movements. Fidgety movements are movements seen in the infant's neck, trunk and limbs continuously in an awake, not fussy baby. They are characterized as small in amplitude, moderate in speed and occur in all directions (38). If fidgety movements are observed, no further GMA assessment is required. If fidgety movements are not observed, a second assessment will be completed at Month 4. The TIMP will be completed at Month 4. The TIMP is an assessment of motor function in infants up to 17 weeks post term (40). The TIMP consists of an Observed Scale of 13 items and an Elicited Scale of 29 items. A trained therapist elicits motor responses by placing the infant in different positions and uses the rating scales to score the quality of responses by choosing the response that best describes the infant's performance. The reliability and validity of the TIMP have been reported in infants 34 weeks postconceptional age through 4 months post term (39).

Infants who have absent fidgety movements or who score below the 16th percentile on the TIMP will be considered at high risk for CP and will progress to Phase 2, the locomotor training phase of the study. These decision criteria are based on a sensitivity of 98% for the GMA (38) and 91% for the TIMP (40) to predict a CP diagnosis. Infants with fidgety movements on the GMA at Month 3 or Month 4 and score above the 16th percentile on the TIMP at Month 4 will not progress to Phase 2. These infants will complete study participation after Month 4 as these infants are unlikely to have CP. Based on the incidence of CP in infants meeting these criteria, we anticipate that 30 infants (50% of those enrolled) will progress to Phase 2.

### 2.7. Phase 2

The intervention phase will begin at Month 5 for infants who continue to Phase 2. Prone locomotor training (Months 5–9) and upright locomotor training (Months 9–18) will be delivered by the research physical therapists trained on each protocol as detailed in the study operations manual. Participants will receive prone locomotor training until 9 months of age, unless they achieve the ability to crawl six feet before 9 months of age. If this occurs, they will move into the upright locomotor training at this time. Participants will receive upright locomotor training until 18 months of age, unless they achieve independent walking. If this occurs, they will end participation at this time. A training log will be maintained for each session. Two sessions per month will be videotaped and reviewed on an ongoing basis to confirm treatment fidelity and for coding behavioral measures.

#### 2.7.1. Prone locomotor training

**Figure 3:**
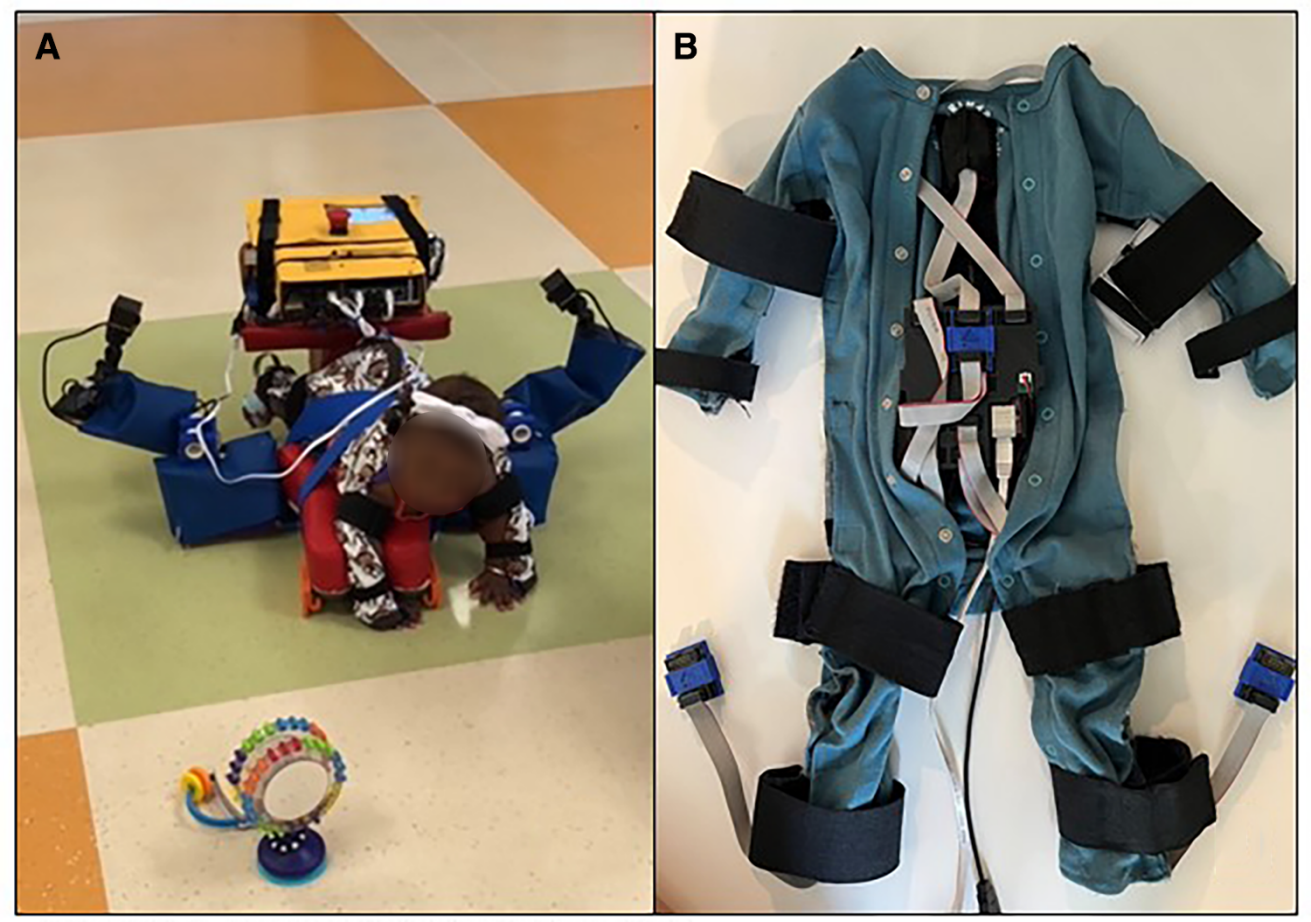
(**A**) SIPPC. (**B**) Motion capture suit.

Prone locomotor training will be delivered using the Self-Initiated Prone Progression Crawler (SIPPC) robot and protocol (Figure 3). These sessions will occur at the infant's home or childcare facility, at their inpatient bedside, or in a Center for Rehabilitation outpatient location, depending on the most convenient feasible location for the family. The SIPPC robotic device (41) consists of two power wheels, a platform mounted to a force/torque sensor, and a motion capture suit with 12 inertial measurement units (IMUs), from which the position of the trunk and limbs is estimated. The infant's attempts to crawl are detected using both the force/torque and the motion capture suit. The SIPPC augments the infant's effort by propelling the infant in the indicated forward or turning direction. The SIPPC is also fitted with three cameras to capture the infant's movement effort and behavior. For each therapy session, the physical therapist gently secures the IMU instrumented suit with straps over the infant's own clothing and places the infant prone on the SIPPC platform. The training protocol is: (1) Warm-up. The infant is given 1–2 min to play with toys and get accustomed to being placed on the SIPPC. We use both familiar and novel toys. (2) Assisted movement of the arms and legs. The therapist or caregiver moves the infant's arms and legs to simulate crawling towards the toys to provide the infant a sense of how to move the device. (3) Calibration of the infant's arm and leg positions. (4) Configuration of the robot interaction. This includes defining the types of information used to trigger assistive movements and setting software-defined thresholds that determine when assistance is triggered. (5) Self-initiated and directed mobility on the SIPPC towards toys or the caregiver for five minutes. Three videotaped 5-minute trials are conducted, within the infant's tolerance, with a repeat of Step 3 before each trial. Total training time is 15 min if the child completes all trials.

#### 2.7.2. Upright locomotor training

**Figure 4:**
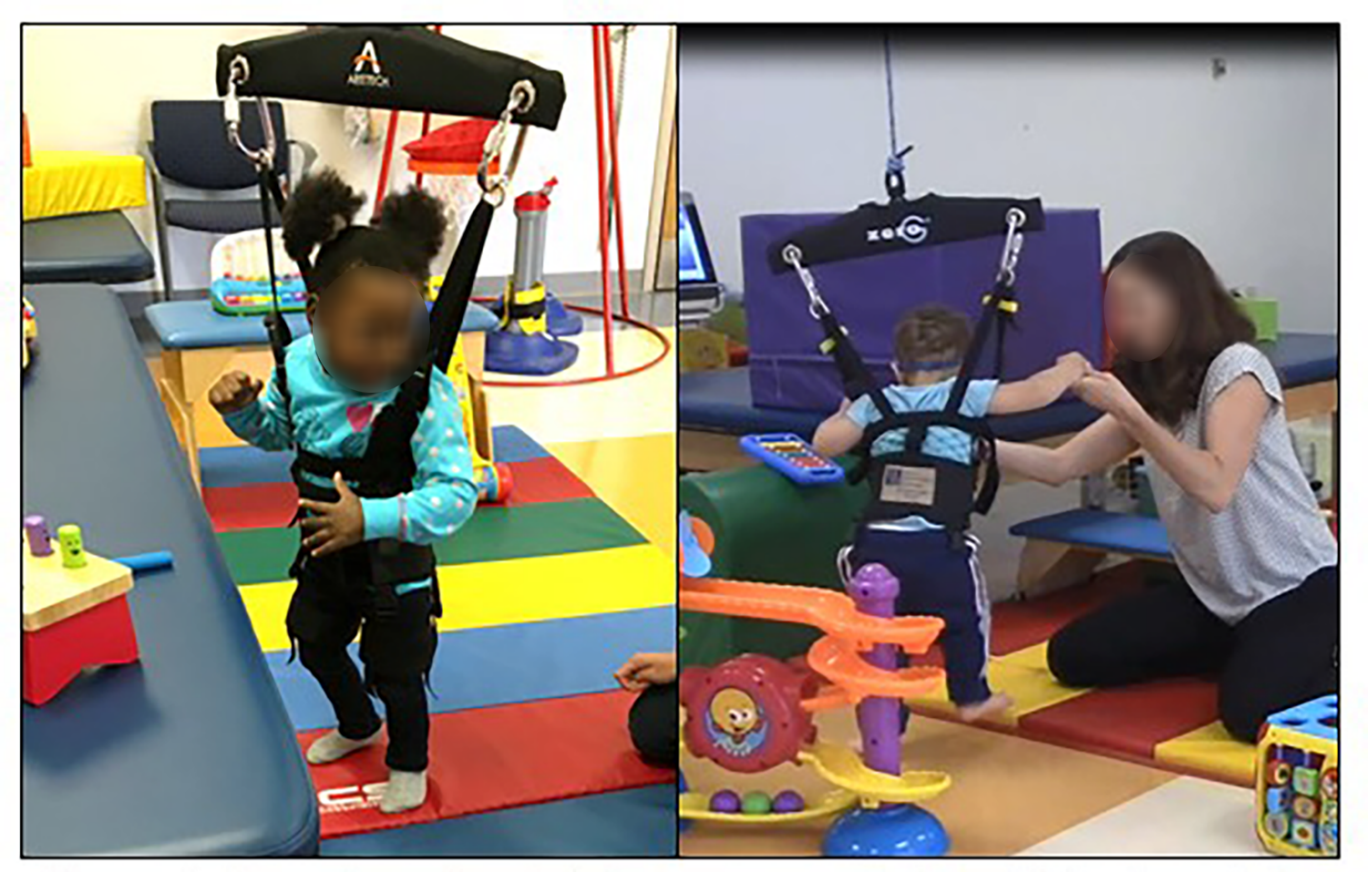
Examples of upright locomotor training with body weight support.

Upright locomotor training sessions will occur at the CHOP main campus. Participants will receive upright locomotion training with dynamic weight support using the ZeroG Gait and Balance training system (Aretech LLC, Ashburn, VA) for each 30-minute therapy session (42). The dynamic weight support system continuously provides a constant amount of weight assistance but does not direct or constrain movement. Initial amount of weight assistance will be 50% of the infant's body weight. Weight assistance will be gradually reduced as postural control and coordination improve, but only when upright locomotion can be performed with the same (not greater) amount of therapist assistance at a lower level of weight support. Participants will be weighed once per month and weight support will be adjusted accordingly. The environment will be arranged to encourage active motor exploration and to promote error-generating experiences and variability in upright activities. The floor area within 3 feet on either side of the overhead track for a distance of 15 feet (approximately 90 square feet total) will be indicated by a thin rubber mat and arranged with pediatric toys to encourage walking, tailored to the child's motor ability and interests. This arrangement works well to keep children within the limits of the overhead track, provides ample space for motor play and exploration, and provides some cushion when the children fall (Figure 4). The therapist will assist the child as needed to encourage upright locomotor activities, but only the minimum amount needed to perform the task (i.e., not more assistance to promote “typical” movement pattern). The therapist's first priority is to encourage positions where the trunk is upright and the hips are extended (such as standing and kneeling). These activities will be varied in context—including taking steps forward, backward, and to each side, stepping on and over different surfaces. The second priority is to encourage dynamic and challenging activities over static and easy activities (such as walking over standing and transitioning to stand without pulling up on a surface over pulling up on a surface). Dynamic and challenging movements are encouraged to maximize error experience with activities that may destabilize the child's postural control and balance. Finally, the therapist will assist the minimal amount required to achieve a success rate of approximately 50% for a particular task. When possible, the child will be encouraged to initiate movements and transitions on his/her own rather than at the facilitation of the therapist. To promote variability, activities will vary frequently as is typical during motor development. The therapist's expertise will be focused on designing a salient and challenging environment for each infant's interests and ability level to encourage error experience, self-initiation, and variability, and on determining the appropriate amount of weight assistance.

### 2.8. Measures

#### 2.8.1. Demographic variables

We will collect relevant variables from the NINDS Common Data Elements (43) (CDEs) for Cerebral Palsy, including: General Demographics, Social Status, Pregnancy & Perinatal History, Medical History, Motor Development History, Neurological Exam, Rehabilitation Therapies, Gross Motor Function Classification System at 18 months of age (3), and the Manual Ability Classification System at 18 months of age (44).

#### 2.8.2. Predictor variables

Training characteristics will be collected during therapy sessions as potential predictor variables for acquisition and retention of locomotor skill, including measures of error, movement index, postural control and movement variability. These will be measured using video coding of recorded therapy sessions using Datavyu video coding software (45). Error during prone locomotor training will be defined as the number of corrective turns generated by the child. Corrective turns are a change in direction less than 2 s following another turning movement. Error during upright locomotor training will be defined as the number of losses of balance. Analyses will be performed with total amount of error, and frequency of error normalized to the distance travelled (error rate). The number and rates of error will be measured using the crawling and walking training robots. Movement index is the percent of time moving during each therapy session. This variable will be calculated using IMU data. Postural control is the percent of time with the head lifted during crawling therapy sessions, and the percent of time spent in an upright posture during walking therapy sessions. This variable will be determined by video coding. Movement variability during crawling is the activities that engage the SIPPC assist mechanism, measured by the IMUs. Movement variability during walking is the number of different motor activities in which the child engages, determined by video coding.

#### 2.8.3. Moderator variables

Measures of early spontaneous leg movement, cognition and motivation to move will be collected as neurobehavioral variables outside of training that we expect to influence outcome. Collection of spontaneous leg movement is described above in Phase 1 section. Analysis of spontaneous leg movement will be performed using existing, validated algorithms (25, 46). Cognition will be measured by the BSID-IV cognitive subscale age equivalent (47) at three time points (Months 5, 9, 18) by a trained assessor. To avoid a learning effect from repeated testing, this will be administered only before and after each type of locomotor training (prone and upright). Motivation to move will be measured by the Motivation to Move scale (MTM) (48) at all six assessment time points and will be used as a potential moderator variable of outcomes. Motivation to move is associated with earlier gains in motor milestones in infants with typical development (48). This rating scale will be scored from 2-minute video recordings of infant behavior in prone during each assessment session.

#### 2.8.4. Locomotor skill

Locomotor function assessments will be completed for infants who progress to Phase 2 (the intervention phase) at six time points (Months 5, 7, 9, 12, 15 and 18). The primary outcome measure for Aim 1 is the Movement Observation Coding System (MOCS), a task-specific measure of locomotor performance. The MOCS uses video coding to assess postural control, arm and leg movements, and goal directed movement. The scale has 27 items with 5 items to assess position of head, trunk, hands and legs in prone and 22 items that assess the effect of the child's arm and leg movements on movement of the SIPPC and prone locomotion. The scale has been validated with infants with various disabilities (49). The primary outcome for Aim 2 is the Gross Motor Function Measure (GMFM), a measure of real-world locomotor capacity (50). Subscore dimension C (crawling and kneeling) will be used to determine change in prone locomotor function after prone training, and subscore dimension E (walking, running, jumping) will be used to determine change in upright locomotor function after upright training. See Table 1 for a summary of predictor, moderator and locomotor skill measures.

**Table 1:** Measures of training predictors, locomotor skill, and moderators.

	Variable	Prone measure (source)	Upright measure (source)
Training predictor	Error (Aim 1): Total amount	Corrective turning (SIPPC)	Losses of balance (Video coding)
	Rate	Corrective turning (SIPPC)/ Distance travelled (SIPPC)	Losses of balance (Video coding)/ Distance travelled (ZeroG)
	Movement index (Aim 2)	Percent of time moving (IMUs)	Percent of time moving (IMUs)
	Postural control (Aim 2)	Percent of time with head up (Video coding)	Percent of time in upright position (Video coding)
	Movement variability (Aim 2)	Number of different movements that engage the assist mechanism (IMUs + SIPPC)	Number of different motor activities (Video coding)
Skill	Task performance (Aim 1)	Movement Observation Coding Scale (Video coding)	Movement Observation Coding Scale (Video coding)
	Locomotor function (Aim 2)	GMFM dimension C (Therapist administration)	GMFM dimension E (Therapist administration)
Moderator	Early spontaneous movement	Quantity, variability, and acceleration of leg movements (IMUs)	
	Cognition	Percentile rank (Bayley Scales of Infant and Toddler Development—Fourth edition, Cognitive subtest)	
	Motivation to move	Motivation to Move (MTM)	

### 2.9. Retention and locomotor training adherence

To promote retention over the extended study duration, we expect the majority of sessions during the first 9 months to be conducted in the participant's home, family scheduling preferences will be met, and caregivers will be compensated. Participants will not be withdrawn from the study for missing treatment sessions. Outcome data will be collected at assessment sessions regardless of adherence to treatment schedule. Of the 30 participants who are expected to progress to the locomotor training phase, we anticipate attrition of 20% by Month 18 resulting in 24 participants with evaluable data.

Given the medically fragile participant sample and the number of study visits during the intervention phase (3 times per week for 13 months), we expect that most infants will miss several sessions and some infants will miss many sessions. We will make up missed sessions within the week when possible. We expect that when participants are sick, we will not be able to make up those sessions. Because most of the prone locomotor training will be conducted in the infants' homes or child-care facilities, we anticipate fewer missed sessions than during upright locomotor training. We will maintain a log of therapy attendance, which may be used as a covariate in the statistical analysis.

Participants may withdraw from the study at any time without prejudice to their care. Intent to treat procedures will be followed such that participants will not be withdrawn from the study by the investigators for lack of adherence to the treatment schedule.

### 2.10. Statistical analysis

The primary endpoint of the study is the Month 18 assessment. This in-depth longitudinal, single cohort study is not powered to achieve specific significance testing, but rather to estimate effect sizes related to the multidimensional complex relationships between training characteristics, locomotor skill, and potential mediating characteristics. The usual descriptive statistics and normality checks will be performed. While the incidence of CP is greater in males than females, and we expect our sample to reflect that, we do not expect sex differences in motor performance (51). We will compare our data between males and females and report differences and similarity. If needed, missing data will be imputed using appropriated method for imputation such as the maximum likelihood method or the multiple imputation method, an iterative form of stochastic imputation of missing data.

#### 2.10.1. Aim 1: characterize patterns of error-based learning during the acquisition of locomotor skill in infants at high risk for CP

The distribution of the variables will be examined to assess the normality and homogeneity of variances. If needed, an appropriate method for variable transformation will be selected and applied to approximate the normal distribution. The measures will be summarized by time point using descriptive statistics. Spaghetti curves will be generated for the variables for each infant to understand the pattern of changes during the crawling and walking phases. Change scores and a matrix of pairwise Spearman correlation coefficients will be examined. The relationship between the repeated measures of task performance during prone and the repeated measures of error during prone training will be explored using the mixed effects modeling approach with random intercepts or generalized estimating equations. A similar analysis will be used for exploring the relationship between the repeated measures of task performance during upright and the repeated measures of error during upright training.

#### 2.10.2. Aim 2: develop a predictive model of locomotor skill retention in infants at high risk for CPp based on training characteristics and moderators

We will examine the relationships between training predictors, neurobehavioral moderators and locomotor skill. The primary dependent variable is locomotor function (GMFM C for crawling and GMFM E for walking). The independent variables are error, movement index, postural control, and movement variability (see Table 1) which are measured repeatedly during training sessions Months 5–18 and will be subjected to data reduction. The moderators are early spontaneous movement behavior (Month 4), cognition (Month 5 for prone and Month 9 for upright), and motivation to move (Month 5 for prone and Month 9 for upright). One model will be developed for prone locomotor skill, and a second for upright locomotor skill. The small sample size and the potential correlation among the independent variables, including the moderators, will require careful examination and interpretation of the regression models' results. The focuses will be on obtaining estimates of slopes and their 95% CIs. We will explore modeling the dependent variable and the repeated measure of the independent variables by utilizing the mixed effects modeling approach. The three moderators may be grouped mean centered (i.e., subtracting group mean value from individual measure). The moderators will be examined individually in the mixed effect models. The moderating effects will be assessed by incorporating as an interaction of moderator X each of the independent variables utilized in the mixed effect model. The magnitude and the sign of the interaction term will be assessed to explore the moderating effects of the proposed moderators. Also, when appropriate, we will employ multivariate cluster analysis algorithms and the linear discriminant analysis to explore the relationship among and between variables. The results will help us to choose the important factors for estimating predicative models linking learning strategies to locomotor skill.

#### 2.10.3. Aim 3: determine locomotor learning strategies in infants at risk for CP that transfer from prone to upright locomotion

We expect to identify critical thresholds of error and other training characteristics from prone locomotor training, above which greater upright locomotor learning is likely. Data processing and reduction will be as described for Aims 1–2. The mixed effects modeling approach with random intercepts and the generalized estimating equations will be utilized in estimating the relationship between walking function (continuous dependent variable GMFM E) and crawling task characteristics (training predictors and task proficiency measured repeatedly over time).

### 2.11. Adverse event reporting/harms

Participant safety will be monitored by maintaining an adverse event log. Stored in REDcap (Research Electronic Data Capture), adverse events will be recorded as serious or not serious, expected or unexpected, and related or unrelated to study participation. Since the study procedures are not greater than minimal risk, serious adverse events are not expected. If any unanticipated problems related to the research involving risks to participants or others happen during this study, they will be reported to the IRB in accordance with local and regulatory requirements. Adverse events that are not serious but that are notable and could involve risks to participants will be summarized in narrative or other format and maintained in the REDcap adverse event log and submitted to IRB at time of continuing review. If the Investigator becomes aware of any serious, related adverse events after the subject completes or withdraws from the study, they will be recorded in the source documents and on the case report form.

### 2.12. Data monitoring

The incidence of adverse events is expected to be low in this minimal risk research, justifying safety monitoring by the PIs and CHOP single IRB of record. The PIs will be responsible for monitoring the safety of all participants. All study procedures will receive IRB approval prior to recruitment or enrollment of participants. Safety procedures and any adverse events will be reviewed and evaluated at each monthly operations meeting with the PIs and clinical team, and at each annual investigators' meeting with the entire study team. Standard procedures for all data collection methods will be reviewed in-person at the start and periodically throughout the study. The PIs will be actively involved in reviewing the raw data of study participants and will bring to the attention of the IRB adverse events and unexpected problems. Unexpected safety concerns will also be communicated with the NIH Program Official in accordance with study regulations.

### 2.13. Data quality

All study procedures will be detailed in a study-specific Clinical Trials Operations Manual. The operations manual will be used to train the study team in Year 1. The research assistant will check all paper source data for completeness before the end of each testing session.

#### 2.13.1. Treatment fidelity & video coding

Adherence to the prone and upright locomotor training protocols will be verified by review of the biweekly videotaped training sessions. Each session will be coded for adherence or non-adherence to the protocol. Therapist retraining will occur if treatment fidelity for any individual therapist falls below 90% of sessions. All video coders will be trained to 85% reliability and training videos will be 20% double coded to ensure that 85% reliability is maintained throughout the course of the study.

#### 2.13.2. Assessment administration reliability

*Test of Infant Motor Performance*. Before administering any Test of Infant Motor Performance (TIMP) assessments, the assessors will reach greater than 85% administration reliability (ICC) with a TIMP developer and less than 5% misfitting items on scoring reliability based on the Infant Motor Performance Scales (IMPS). All TIMP assessments will be videotaped. Every 10th assessment will be checked to assure reliability is maintained at >85%. If less than 85% agreement is observed, additional videos will be examined to determine if it was a systematic scoring error. *Gross Motor Function Measure*. Before administering any Gross Motor Function Measure (GMFM) assessments, the testers will reach greater than 90% reliability (ICC 0.945) with expert consensus opinion across 10 videos of a range of GMFM scores. All GMFM assessments will be videotaped. One assessment will be randomly checked every 6 months to assure reliability is maintained at greater than 90%. If less than 90% agreement is observed, additional videos will be examined to determine if it was a systematic scoring error. *Bayley Scales of Infant and Toddler Development*. Before administering any Bayley Scales of Infant and Toddler Development (version 4) cognitive subscale assessments, the testers will reach greater than 85% reliability (ICC) with the National Pediatric Rehabilitation Resource Center (C-PROGRESS) training/reliability program. All Bayley assessments will be videotaped. One assessment will be randomly checked every 6 months to assure reliability is maintained at greater than 85%. If less than 85% agreement is observed, additional videos will be examined to determine if it was a systematic scoring error.

## 3. Discussion

Motor development is sequential with early skills serving as a substrate for subsequent ones (52, 53). However, evidence suggests that for children with CP, this sequence is unpredictable. The underlying source for this developmental discontinuity is not understood, yet such information is crucial to the development of earlier and effective interventions. Findings from intervention studies with this population highlight the importance of retention and transfer of movement learning strategies (7–10). Our preliminary findings have shown that infants at risk for CP, like typically developing infants, are capable of using reinforcement-based feedback from robots to engage in task-specific learning, but unlike their typically developing peers, the transfer of the learning strategies from one context to another is inconsistent. Our prone and upright locomotion pilot studies have focused on one skill and were time limited. We will examine if motor behavior or training characteristics during prone locomotion predict upright locomotor learning.

In addition, a central problem in predicting functional outcomes is the multidimensionality of CP, including severity of motor impairment and the extent of secondary sequelae such as cognitive impairment. Locomotor learning is compounded by these factors and by developmental and neural immaturity. Locomotion expands infants' motor activities beyond sitting and reaching to include movement within their environments. Not only are infants learning muscle activation and selection of movement patterns to travel to a target, but other developmental systems such as spatial cognition, memory, and social emotional processes are positively impacted as well (52, 54). Despite their significance to development, to date no models exist about how infants at risk for CP learn to crawl or walk, which will be a goal for this investigation.

This clinical trial will provide valuable information about the way infants at risk for CP learn to move and give insight to the moderators that may affect whether a child with CP does or does not learn to locomote independently. Learning how infants retain and transfer learned movement strategies to other locomotor skills is an important tool that can be integrated into the current standard of care practice for children with CP. This understanding will contribute to the development of interventions that improve independent mobility and participation in infants with CP and lead to greater physical function over the lifetime.

## 4. Ethics and dissemination

This study has been approved by the IRB of CHOP, which has been acknowledged by all participating sites as the IRB of record. The study will be conducted in full accordance with all applicable CHOP Research Policies and Procedures, and all applicable Federal and state laws and regulations. The investigators will perform the study in accordance with this protocol, obtain consent, and report unanticipated problems involving risks to subjects or others in accordance with The Children's Hospital of Philadelphia IRB Policies and Procedures and all federal requirements. Data collection, recording, and reporting will be accurate and will ensure the privacy, health, and welfare of research subjects during and after the study. Any protocol modifications will require approval from the CHOP IRB and will be communicated to all investigators and participants. The ClinicalTrials.gov website will also be updated with any significant protocol modifications.

### 4.1. Informed consent

A parent or legal representative of each potential participant will receive a verbal and written explanation of the purposes, procedures and risks of the study. If they are interested, eligibility will be screened by the research physical therapist, the study coordinator, or an investigator. Informed consent will be obtained by study staff from the N/IICU, the research physical therapist, the study coordinator, or an investigator, and will be documented in the electronic database. The family will have the opportunity to ask questions throughout the entire process. A parent or legal representative must provide written informed consent prior to the start of any study activities. Written assents of minors will not be obtained due to the age of the participants. Caregivers will also be asked for their permission to share videos with other authorized Databrary users (55). Not agreeing to share videos on Databrary will not preclude enrollment in the study, and those videos will remain privately archived for the study team.

### 4.2. Dissemination

We will publish our findings in scientific journals in the fields of pediatrics, rehabilitation, computer science, engineering, infant development, neonatology and neurology. We will often publish in open-access journals to allow free access to this federally funded work. Any financial or competing interests will be disclosed in each publication but currently no study investigators have any conflicts of interest to report. Determination for authorship for each publication will be determined on a case-by-case basis by the PI's while using the authorship guidelines of the publishing journals. Professional writers will not be utilized in the dissemination of study results and the funding agency will not have a role in the decision to submit for publication.

## Data Availability

The original contributions presented in the study are included in the article/Supplementary Material, further inquiries can be directed to the corresponding author/s.
